# The role of social identity in a suicide prevention programme for construction workers in Australia

**DOI:** 10.1093/heapro/daae140

**Published:** 2024-10-22

**Authors:** Jorgen Gullestrup, Samantha Thomas, Tania King, Anthony D LaMontagne

**Affiliations:** Institute for Health Transformation, Faculty of Health, Deakin University, 1 Gheringhap Street Geelong Victoria 3220Australia; Institute for Health Transformation, Faculty of Health, Deakin University, 1 Gheringhap Street Geelong Victoria 3220Australia; School of Population and Global Health, University of Melbourne, University of Melbourne, Victoria 3010Australia; Institute for Health Transformation, Faculty of Health, Deakin University, 1 Gheringhap Street Geelong Victoria 3220Australia; School of Population and Global Health, University of Melbourne, University of Melbourne, Victoria 3010Australia

**Keywords:** Mental health, suicide prevention, lived experience, peer support, MATES in Construction

## Abstract

Each year, more than 700 000 people die by suicide globally, the majority of whom are men. The United Nations and World Health Organization have set targets to reduce suicide rates by one-third by 2030. While large-scale suicide prevention programmes are required to meet these targets, diffusion of these types of initiatives is difficult—particularly with male populations. This qualitative study investigated the MATES in Construction suicide prevention programme in Australia. Guided by Social Identity Theory and the Social Identity Model for Collective Action, the study aimed to understand why construction workers chose to volunteer and advocate for industry-based suicide prevention programmes, and how their worker identity, solidarity and relationships impacted their volunteering and advocacy. Semi-structured interviews were conducted with 28 participants who had chosen to engage with MATES as volunteers. Data were interpreted using a reflexive approach to thematic analysis, and four themes were constructed from the data relating to feelings of belonging, connection and solidarity between workers and their industry; how specific context and roles impacted identity while existing within an overall sense of identity and solidarity; how industry mateship supported engagement in suicide prevention; and how the role of lived experience, mateship and responsibility provided hope for change. Providing intervention skills to workers, particularly workers with a lived experience of mental ill-health, empowered them to believe that they could make a difference by acting collectively. The MATES engagement model described in this study may have applications for other health promotion prevention programmes targeting male cultures.

**Contribution to Health Promotion**

- Sustained, large-scale programmes are important in meeting targets for suicide prevention in male populations.
- Social identity may be an important factor in continued engagement in suicide prevention programmes in the predominantly male construction industry.
- Strong group identity generated feelings of mateship and solidarity between workers and the industry, including a sense of duty to engage in suicide prevention activities.
- Lived experience of poor mental health created hope and purpose in acting collectively to address the risks of suicide.
- Group identity and belonging is a key attribute that should be considered in the development of public health programmes targeting predominantly male cultures.

## INTRODUCTION

### Suicide as a public health issue

The World Health Organization (WHO) estimates that globally more than 700 000 people die by suicide each year (WHO, 2021b). The United Nations (UN) has set a Global Sustainable Development Goal to reduce premature mortality from non-communicable diseases by one-third before 2030, which requires an increased focus on the prevention, treatment, and promotion of mental health and well-being (United Nations, 2015). Suicide is a key indicator for this goal and the WHO has prioritized suicide prevention (WHO, 2021b). A significant factor in suicide risk is employment status. Being unemployed is generally associated with elevated risks of suicide compared to those employed (Milner *et al*., 2014b; Llamocca *et al*., 2023). It should be noted that although suicide rates may be higher amongst the unemployed, this group is much smaller than the employed population and in absolute terms there are many more suicides amongst employed workers (Milner *et al*., 2014a). The nature of work also impacts suicide rates. For example, employment in manual and blue-collar trades has been associated with higher suicide risk compared to white-collar workers (Milner *et al*., 2013). Men are generally at significantly higher risk of suicide than women (Bandara *et al*., 2024) and male construction workers in Australia have been identified as having suicide rates as high as twice that of other employed Australian men (Maheen *et al*., 2022). It should be noted that research has indicated a narrowing of the gap in suicide rates between Australian construction workers and other employed men in Australia since 2007 when the MATES programme was introduced - although no causal link has been established (Doran *et al*., 2016; Martin *et al*., 2016; Maheen *et al*., 2022). While much of the world has made progress towards the UN Sustainable Development Goal, suicide rates in many industrialized countries including the United States and Australia have either remained unchanged or even increased (Dattani *et al*., 2023). In 2021, the WHO issued the ‘LIVE LIFE implementation guide for suicide prevention in countries’ (WHO, 2021a). One example featured as best practice in the WHO guide is the Australian workplace suicide prevention programme MATES in Construction (MATES).

### The MATES in Construction programme

MATES is an industry-based suicide prevention programme that targets workers in predominantly male industries. It operates in Australia and New Zealand with programmes in the construction, mining, energy and manufacturing industries (MATES in Construction, 2024). MATES is a collaboration between employers and unions and aims to engage workers in improving mental health and reducing suicide (Neis and Neil, 2020). The programme does this by raising awareness of suicide across the targeted industry; providing skills and tools for workers locally and thus creating more resilient workplaces; connecting workers in distress to relevant help and support; and finally, through a feedback loop in partnership with researchers to the industry for continual improvement (Gullestrup *et al*., 2011; WHO, 2021a). A recent systematic review of the MATES programme found evidence for the effectiveness of the MATES programme in improving mental health and suicide prevention literacy, improving helping intentions and reducing stigma (Gullestrup *et al*., 2023). The review highlighted the importance of worker-to-worker peer approaches in suicide prevention, such as MATES, particularly in predominantly male industries.

### Diffusion of suicide prevention programmes

Diffusion of public health prevention programmes to larger and male-dominated populations is notoriously difficult. Rogers (2002, p. 990) defines diffusion in public health prevention as ‘*the process through which an innovation is communicated through certain channels over time among the members of a social system*’. Suicide is a serious and yet relatively rare event, even amongst high-risk populations. To achieve diffusion of a suicide prevention programme, individuals must accept that action is required to prevent a relatively rare, intangible but unwanted consequence that may or may not occur at some point in the future. In the first published evaluation of MATES, the relatively rapid diffusion to engage more than 7000 workers was noted as demonstrating the social validity of the programme (Gullestrup *et al*., 2011). Fourteen years later, the MATES website indicates 315 320 construction workers had been trained and more than 34 000 volunteer workers engaged in skills training to assist, across an industry employing approximately 1.3 million workers (Australian Government., 2023; MATES in Construction, 2024). MATES was initially developed for, and implemented in, the large commercial, residential and civil sectors of the industry with high levels of unionization, but has since spread to smaller project and non-unionized parts of the industry.

**Fig. 1:**
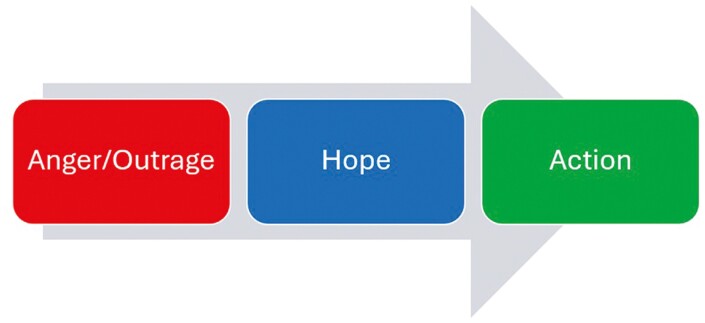
Anger/outrage, hope, action model illustrated based on LaMontagne and Shann (2020).

The substantial diffusion of the programme across the construction industry suggests it could be worth investigating why construction workers choose to volunteer and advocate for the MATES programme (Gullestrup *et al*., 2023). Previous studies into conditions facilitating engagement with another Australian construction industry suicide prevention programme, ‘the Blue Hats’, have described how worker knowledge, skills and abilities can translate into support for colleagues (King *et al*., 2023). Another study has shown the importance of awareness of suicide and ease of engaging with the MATES programme as important motivators for worker engagement (Ross *et al*., 2019). The MATES programme utilizes an *anger/outrage, hope, action* model for engagement (Figure 1), described in the MATES programme logic model (LaMontagne and Shann, 2020). The programme speaks to workers’ identity as workers in a specific industry. It presents high suicide rates as an injustice to workers, and this generates *anger/outrage*. The MATES programme is presented as an effective way to address this injustice collectively and this generates *hope*. It is presumed that this anger and hope will motivate workers to actively engage in suicide prevention in their industry—*action* (LaMontagne and Shann, 2020).

## SOCIAL IDENTITY THEORY

**Fig. 2:**
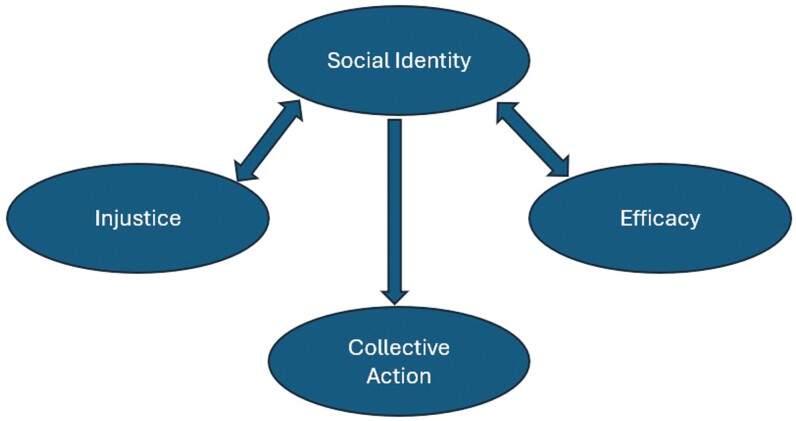
SIMCA model illustrated based on Van Zomeren *et al*. (2008).

Social Identity Theory was originally developed by Henri Tajfel to understand prejudice, discrimination and intergroup conflict in society (Hogg, 2016). The theory considers how basic human motivations and cognitive processes are impacted by individuals’ social context. A social identity arises through feelings of belonging to a group where emotional and value significance is ascribed by the person to this group membership. Social identity provides a reference point for joint values and group norms for the ‘in-group’ and a contrast point to those ‘out-groups’ who do not share the identity. Social identity is central to motivate individuals to form coalitions for social change (Van Zomeren *et al*., 2008). The Social Identity Model for Collective Action (SIMCA) is a model for collective action built from Social Identity Theory. The model (Figure 2) incorporates three socio-psychological perspectives on collective action—*injustice, efficacy and identity* (Van Zomeren *et al*., 2008). Although SIMCA was not explicitly used in the design of the MATES programme, it provides a useful exploratory theoretical framework for qualitative research, which aims to understand construction worker engagement in the MATES programme. Given that suicide prevention programmes are rarely translated and sustained in policy and practice (Krishnamoorthy *et al*., 2024), it is important to understand the range of factors that may stimulate long-term engagement in these types of interventions, and their potential to be diffused to other contexts and settings (Dearing, 2009).

This study was guided by three research questions:

RQ1: Why do construction workers volunteer and advocate for the MATES programme?

RQ2: How do construction workers’ identity and culture impact how they perceive and accept high suicide rates and poor mental health in their industry?

RQ3: How do construction workers' relationships with co-workers and personal experiences impact their willingness to volunteer within, and advocate for, the MATES programme?

## METHODOLOGY

### Approach

This study utilized a reflexive, ‘Big Q’ interpretivist approach to thematic analysis, whereby thematic coding involves an inherently subjective engagement with the data (Braun and Clarke, 2022, 2024b). Qualitative public health research is interpretive and experiential in approach, and has an aim of understanding people’s experiences in the contexts of their own lives, exploring participants ‘subjective experiences and sense-making’ (Braun and Clarke, 2021, p. 3). Big Q research acknowledges that knowledge production is ‘partial, situated and contextual’ and values researcher subjectivity as a resource for the research (Braun and Clarke, 2024a, p. 2). Our approach to this study was also informed by our positionality. It is important to recognize the study was shaped by lived experience, as well as our social and political values. In particular, J.G.’s lived and living experiences of mental health crisis and suicidality informed this study, as well as his role as a former construction industry worker, trade unionist and a co-founder of the MATES programme. These experiences were central in the development and design of this study. J.G.’s ‘insider’ perspective helped to create shared meaning and trust with the participants in this study, which also enabled supportive and ‘knowing’ conversations about the MATES programme. Reflexive discussions with co-authors T.K. and A.D.L. (experts in workplace suicides prevention programmes), and S.T. (a public health sociologist with experience in mental health and addiction research) were centred around public health values of social justice, empowerment and advocacy (Charmaz, 2017). Reflexive discussions were not aimed at tackling any perceived ‘bias’ or ‘misemphasis’ as a result of researcher positionality. Our reflexive process involved regular meetings in which we discussed our assumptions and choices, and sought to challenge our own thinking (through the use of theory and published literature and the narratives of the participants).

The study was approved by the Deakin University Human Research Ethics Committee (2021-406).

### Criteria for recruitment

The study recruited individuals over the age of 18 and living in the Australian state of Queensland who had chosen to engage with the MATES programme in the construction industry as a volunteer, facilitating or supporting the programme. Queensland was selected because it was the Australian state where the MATES programme was established and has had the greatest reach within the Construction industry. In order to recruit participants, a range of convenience and purposive approaches were used. Initially, a sample of six participants were recruited through MATES staff. MATES staff provided information about the research directly to potential participants who met specific profiles. This ensured individuals with a range of different industry roles and interaction opportunities with MATES were invited to participate. This initial group included workers, managers, industry leaders, policy makers and researchers. Interested individuals then registered their interest with the research team through an online registration form to ensure that confidentiality was respected. Workplaces were not given information about who registered interest in the study or participated. Reflection following these interviews led to a decision to focus further recruitment on on-site workers and leaders. Recruitment continued via social media, word of mouth and through MATES field staff distributing information about the project on construction sites. Participants registered their interest through an online form.

Following the completion of the first 15 interviews, it was decided that further recruitment should target blue-collar workers directly. This was due to an overrepresentation of participants from white-collar and management roles. All participation was voluntary, and no financial incentives or compensation were provided.

## DATA COLLECTION

All interviews were conducted by J.G., with S.T. participating in one interview to engage in reflexive practice about the interview schedule. Data were collected between January 2022 and February 2024 through semi-structured online interviews between 31 and 77 min in duration (mean = 49 min). Interviews were video recorded with participants permission and the transcripts were transcribed either by the first author or a professional transcription company. All data was anonymized. Participants were provided with copies of the transcript from their interview. While participants were offered the opportunity for further feedback on the interview, no participants took up this opportunity.

At the start of the interview a range of socio-demographic questions were asked including age, length of time in the industry, personal identities important to them such as gender, sexual orientation, cultural or linguistically diverse backgrounds, highest level of completed education and whether they identified as First Nations Australians. Participants were then asked questions in relation to this study across four topics of investigation:

- The MATES programme: How they would describe the programme to a person who did not know it, what they found useful about it and what they felt could be improved.
- Engagement in the MATES programme: Their personal role in the programme and what other roles they were aware of. They were also asked what role they would like to play and how they felt about the role they were currently playing in the programme.
- Mateship in the industry: How they understood mateship and their personal experiences with mateship. Participants were further asked whether they felt mateship was a feature of the construction industry and if they felt there was a specific construction industry type of mateship.
- Participant lived experience: Participant were offered opportunity to talk about their own personal lived experience of mental health crisis and suicidality. It was explained that the question was entirely voluntary and that they did not have to answer it if they did not feel it was appropriate. This question was included as previous studies have shown the importance of lived experience in taking on volunteering roles within the programme (Ross *et al*., 2019).

## DATA ANALYSIS

Data analysis followed the six-step approach to reflexive thematic analysis (RTA) (Braun and Clarke, 2021). Importantly, this was not a linear process, but a process of back and forth occurring between these steps. The first step involved familiarization with the data. All transcripts were read and reviewed in detail by J.G. to become familiar with the material. Co-authors were also provided with transcripts and had the chance to read and reflect on the interviews. Notes were taken documenting any initial thoughts about the interviews in relation to the theoretical concept of identity and the research questions. The research team had regular meetings and discussions about the early impressions from the data and initial notes were made. This also informed any changes to subsequent interviews, including areas to prompt, and any new concepts to explore. J.G. then started to code data segments, with a particular focus on data segments that could capture insights into the research questions. Data were coded for both semantic and latent meaning, with the initial codes discussed with the team. Journalling allowed for reflection on the different concepts in the data. The coding of the data was both deductive (driven by the researchers existing experiences and views, and concepts from Social Identity Theory), and inductive (from the participants own experiences). The most time intensive step involved iterations of generating initial themes (step 3), reviewing themes (step 4) and defining and naming themes (step 5). Codes were initially grouped by J.G. into preliminary themes and subthemes. Short abstracts were written about each theme to ensure that they were cohesive, had clear boundaries and told an overall story. We discussed any differences in interpretations and perspectives, and constructed shared understandings of meaning from the data. We developed three themes that were presented in the initial paper that was submitted for peer review. After peer reviewer comments about the cohesiveness of the themes, we revised the themes again with a focus on simplifying themes. We developed four themes, each with a smaller number of concepts and subthemes. The writing-up of findings (step 6) was iterative—including through the peer review process.

## RESULTS

### Participant characteristics

Table 1 provides an overview of the general characteristics of participants. A total of *n* = 28 individuals were interviewed for the study. Participants were aged 29–57 years (mean = 45). The majority of participants (*n* = 26) identified as male. The length of experience in the industry varied from 4 years to 42 years (mean = 22), with formal education ranging between finishing grade 9 to a PhD. Most of the participants identified as having a lived experience of mental ill-health and/or suicidality (*N* = 27).

**Table 1:** Participants characteristics

Gender	Male *N* = 26 Female *N* = 2	
Age	18–29 Years *N* = 1 30–39 Years *N* = 5 40–49 Years *N* = 11 50–59 Years *N* = 11	Mean *M* = 44 years old
Experience in construction industry^a^ (two workers from the energy industry excluded)	1–9 Years *N* = 1 10–19 Years *N* = 10 20–29 Years *N* = 11 30–39 Years *N* = 4 40 Years or more *N* = 2	Mean *M* = 22 years in the construction industry
Highest complete level of education^a^	PhD (AQF 10) *N* = 1 Masters (AQF 9) *N* = 2 Bachelor (AQF 7) *N* = 5 Advanced Diploma (AQF 6) *N* = 1 Diploma (AQF 5) *N* = 3 Certificate IV (Advanced Trade AQF 4) *N* = 6 Certificate III (Trade AQF 3) *N* = 6 High School Grade 12 (AQF 1) *N* = 1 High School Grade 9 (AQF 1) *N* = 1 (AQF—Australian Qualification Framework)	Mean *M* = 5 (Bachelor level)
Role in the industry (more than one role possible)	MATES Staff (Field Officer) *N* = 2 Construction Worker *N* = 14 Supervisor/Manager *N* = 10 Connector^b^*N* = 15 ASIST Worker^b^*N* = 4 Industry Leader *N* = 2 Policy Maker *N* = 2 Researcher *N* = 1 Other—Union Delegate *N* = 9 Other—Health and Safety Representative *N* = 6	
Significant identities^c^	LGBTI+ *N* = 0 CALD *N* = 0 Aboriginal/Torres Strait Islander *N* = 2 Mauri (NZ First Nation) *N* = 2	

^a^Two workers from the energy industry excluded.

^b^A specific volunteer role within the MATES programme.

^c^This question was only asked of the last 15 interviewees.

Four qualitative themes were constructed from the data. Examples of the data used to construct these themes are available as Supplementary Material to this article.

### Theme one: a sense of belonging, connection and solidarity with the industry and colleagues

Participants had a strong sense of belonging to the construction industry, with many identifying an ‘invisible bond’ between them across employers, trades and projects. Within this overarching construction industry identity workers also felt connection through sub-identities based on their positionality within the industry. A sense of belonging to the construction industry provided workers with pride and identity. Workers spoke with fondness about past projects and explained that they would ‘check in’ on past jobs when they drove past them or even show them off to friends and families:

*As I say, I’m very much industry, nearly half my life has been the construction industry.* (Field Officer, Male)

*My wife hates it every time we drive past a job I’ve worked on. I just got to let her know.* (Union Delegate, Male)

Participants described that employment in the industry often involved long working hours, working away from home and difficult physical conditions. This added to the identity of being a ‘rough’ construction worker who embraced masculine values and norms. The short-term nature of project-based employment in the industry, meant that connectivity to others was important as these connections often help individuals find their next job.

*It is not a big happy family, but it is a camaraderie thing. Everyone is looking out for each other. If you know someone who needs work, everyone will go out of their way to get someone a job. I think the MATES stuff adds to that.* (Plumber/Union Delegate, Male)

Past jobs served as icebreakers between workers, among whom new relationships were formed by an exchange of work history and common industry-based connections. Having worked on iconic jobs or with high profile workers in the industry created a higher-level intragroup status. The itinerant nature of employment in the industry created a spiderweb of interconnectivity between workers, which participants described as bonding workers even before they met. There was a strong sense of solidarity between workers across the industry, among both those that are personally known and those that are yet to be met. This sense of solidarity and having each other’s backs, particularly for union workers, also led to a strong history of advocating for better safety standards and working conditions. This included a range of bi-partisan industry-wide institutions providing key benefits for workers such as training, superannuation, redundancy, long-service leave, portable sick leave and income protection.

*I think there definitely is a strong camaraderie in our industry. We spend so much time together, more time than we spend with our family at home. It makes it worthwhile getting to know the people onsite, to understand what they do and go through.* (Dogman, Male)

The itinerant nature of work meant that as workers moved from project to project, across different employers, culture and industry norms were continually moderated and negotiated, creating industry-wide social norms and rules. The connectivity of workers across the industry was central to the diffusion of the MATES programme as workers continually advocated for the programme across the industry as they moved from worksite to worksite. Because of this, the embedded nature of the MATES programme as an industry-based programme that was trusted no matter where participants were working was apparent.

*What are we trying to achieve – we are trying to achieve connection with people. Ten field officers in Queensland across hundreds of jobs is the same as me being one safety person across 10 jobs. We don’t have a chance if we rely on us to be the difference but if we can change behaviour and culture by empowering people, then our reach and effectiveness is much greater. The ten MATES field officers on their own is nothing, but with this process it can become 1000’s*. (HSEQ Manager, Male)

One of the benefits of MATES that was highlighted by participants was that it was supported across the construction industry by both unions and employers, and that it was universal across the industry allowing workers to engage with the same programme regardless of which employer they were working with. Participants discussed that industry-wide solidarity was particularly pronounced around significant accidents or events. For example, when a worker had been hurt or killed through an accident at work, the news travelled through the industry network almost instantaneously, and became a mechanism for immediate support both for the worker and their family members:

*When the young 17-year-old form worker died recently. We didn’t know him. He worked on a job site I’d never been on, never seen, except on the news, and he died, and not only did we rally around and support the family, but we also actually marched to change the laws for someone dying. In our industry, we don’t even know people and we come together to support and look after them – their mental health, their sickness and the rest of it. I have never worked anywhere in an industry like this. That is probably why I call it my industry, my job, because we really do look out for one another.* (Union Delegate, Female)

MATES was seen as an industry insider and is therefore trusted by the industry to be able to understand workers and to stay the course where outside programmes may come and go. This featured strongly in explanations of why participants had chosen to trust and volunteer with MATES. It was not only important that the MATES organization was seen as a construction organization, but the peer-based nature of the MATES programme was essential for trust in the programme. Participants expressed a high level of trust and confidence in each other and understanding from a peer was valued higher than professional counselling skills:

*This is our mental health program. The EAP (Employee Assistance Program) just don’t cut it. We have an EAP, everyone has an EAP, but it is not the same as ‘Billy is the MATES person on this site, go and talk to him.’* (Health, Safety, Environment Manager, Male)

### Theme two: While identities could change between work roles and context, there was an overall sense of identity and solidarity across the sector with MATES

While an overall construction industry identity existed, worker sub-identities were also common, with participants moving in and out of different identities depending on different workplace situations. Workers compared and contrasted themselves between in- and out-groups, leading to both one construction industry social identity and several sub-identities existing simultaneously. Some of the more noticeable sub-identities were the distinctions between white-collar and blue-collar workers, between unionized and non-unionized workers, between commercial/industrial and residential construction workers, between blue-collar structural, finishing and services trades. However, even across the sub-identities there was an overall strong sense of solidarity whereby workers, for example, would highlight that MATES needed to expand to other sectors of the industry where they perceived the need was higher:

*Without a doubt it is great that MATES in Construction are doing what they’re doing, they’re doing it very well. They’re doing it in the mining industry, they’re doing it in the tier one* [tier one = generally larger and unionised construction sites] *construction industry. But there’s a whole other area that are probably more at risk and that’s in the domestic sector and the tier two jobs* [smaller jobs with less union presence] *where there’s no union. That’s where I think the next direction needs to go.* (Carpenter, Male)

Several workers highlighted that globalization had meant that the industry had structurally changed from local town or state-based companies to national and international companies dominating medium- and large-scale construction. For example, older workers remembered times where the carpenter became the local builder that grew bigger, but they now saw that management recruited directly from universities to work for companies where the owners were shareholders far removed from the local community. One of the impacts on worker identity was that this meant that some workers no longer saw themselves reflected in some construction leadership structures. They perceived that this was gradually putting distance between business and workers with industry outsiders taking over leadership roles. While MATES was predominately identified with reference to the singular construction industry social identity, and participants identified MATES as being part of *their* industry, the way they chose to engage with the programme differed depending on their position and identity within their specific companies:

*I’m working for quite a big worldwide company now and there is the us and them, between the construction workers, the management team, and the office staff. As much as they like to say there isn’t, there is. But you know when you walk through the office, you can feel the tension, it’s not the same as what we have. They’ve got these beautiful, air-conditioned offices they sit in all day, with snacks provided and drinks in the fridge. But they don’t seem to connect the same as what we do roughing it out you know.* (Union Delegate, Male)

### Theme three: the role of industry mateship in engaging in suicide prevention

Mateship in the construction industry is an industry cultural value and provides social rules for interactions between workers but also social rights and obligations. The concept of mateship was strongly articulated amongst blue-collar workers with both white-collar and blue-collar workers identifying a glass ceiling for mateship between workers and management. While Australian popular culture has strong stereotypes of mateship based on our convict past and wartime ANZAC legends, the mateship identified in this study was not anchored in distant history but current working conditions including the joint and ongoing struggles to improve working conditions.

*There is mateship in the industry, and it is through a familyship of struggle. It is like soldiers. I was in the Army. When you put the green uniform on you are a comrade. The construction industry is like comrades in arms. If you go into a pub and someone says, ‘I worked somewhere…’ you quickly spark up a conversation.* (Safety/Logistics Manager, Male)

It is through mateship that interpersonal social rules within the construction industry are applied. Participants discussed how mateship allowed a certain tone in conversations, including when this tone needed to change, which conversations can and should be had, and which should not. The exact nature of mateship was contextual to the sub-identities of the workforce, power and personal relationship between workers. Mateship in the industry was described as both casual and almost incidental, but also as unconditionally mutual and reliable. It created a casual and flexible tone in the workplace while also knowledge of mutual strength and trust:

*If someone comes in and goes ‘Morning Dickheads’ you know it is ok because we are mates. But we can also sense when something is wrong with somebody. If somebody doesn’t call you a Dickhead it is time to say, ‘let’s have a chat’. It is just something that has evolved. It is a mateship, and it keeps morale high.* (Carpenter, Male)

The MATES mode of engagement was consistent with this model of informality and obligations, particularly through the collective General Awareness Training (GAT), which highlighted the issue of suicide and the MATES model of peer-to-peer support. During the training, workers collectively agree, by a show of hands, that suicide is a significant issue in the industry, that they commit to look out for each other, and volunteer for the programme. Participants described the value of the GAT session in embedding the collective MATES objectives into the mateship culture on-site. This mix of existing mateship industry culture as repurposed by the MATES programme for suicide prevention was a key reason why participants stated that they volunteered with MATES:

*I think the GAT… it does create Awareness, but it also gives opportunity to reflect. I think without the GAT we would not get to the Connectors* [A MATES program Volunteer]*. If we just asked who wants to be a Connector for people in suicidal crisis, people would most likely not come forward, but the GAT makes the case for the rest of the program on site.* (Health, Safety, Environment and Quality Manager, Male)

The concept of belonging to a similar social class also featured strongly in participants responses about mateship. Having similar social class backgrounds created bonds and unity between workers. Workers saw the class background and their blue-collar identity unified in an ongoing struggle for rights, justice and equity. They referenced their own group norms and unity against those who did not share their background seeing their mateship as superior to that of other classes:

*I think it is the people, the people that are drawn to the construction industry tend to be a certain type of person, a blue-collar worker that comes from a lower socioeconomic background. I think that lower socioeconomic background people have a greater sense of community and stick together a bit more, we are all in the same boat type of attitude.* (Tiler, Male)

The underpinning values of mateship and trade unionism were closely aligned in participants’ responses. Trade union language of ‘comrade’ and ‘brother’ was seen as building stronger connection between workers. Application of trade unionism and the MATES in construction programme had strong similarities in that both Unions and MATES are authorized on-site through a collective mandate from the workforce. Both Unions and MATES reached the broader workforce through a site-based peer-network. Both Unions and MATES are industry-wide and industry-based structures supporting these site-based networks. In this context, it was not surprising that many participants in the study who were Union delegates found their roles as delegates for the Union and Connectors (on-site volunteers acting as connecting points between workers in distress and support resources) for MATES highly compatible and at times interchangeable. Delegates found they were better Connectors because they had the trust of the workforce and better Union delegates because the MATES programme enhanced their interpersonal skills.

*It is all the same sort of stuff in the end, the camaraderie, the union, the MATES in Construction thing, it is everyone looking out for each other, getting involved. It is all the same sort of thing which I really do like about Construction. I like that more than plumbing.* (Plumber/Union Delegate, Male)

### Theme four: lived experiences combined with mateship and collective responsibility provided hope for change

The majority of participants in the study identified lived experience of poor mental health and suicide as being part of their reason to engage with the MATES programme. Experiences varied widely from those who were personally impacted as suicide survivors or by diagnosed mental health conditions, those who had lost friends or family members to suicide, those who had experienced the impact of suicide on others, and those who had experienced the impact of suicide on their workplace. While the nature of these experiences varied greatly, the common thread was that participants’ experiences had empowered them with the knowledge that they could, and had to, be part of making a change for the future. Participants described that they could be an example to others, but also that their personal experience would make them more relatable and empathetic towards others going through personal struggle. The value of peer support was also experienced through this lived experience and workers talked about giving back now that they found themselves in a better space:

*I guess that is why the program resonates with me personally, but also why I think it resonates with the industry. When we talk about strengths of the program and why it resonates and the language and all that, but at its essence it is about empowerment. If we can empower people, then they will do the right things whether it is to use the grinder correctly and stay physically safe or whether it is having a conversation with someone struggling and connecting them to help. If we empower people, they will do it.* (Health, Safety, Environment and Quality Manager, Male)

While participants’ strong sense of joint identity as construction workers gave rise to a culture of mateship, and while this bond of mateship meant that the industry’s high suicide rates were seen as an injustice to be fought collectively, it was the lived experience of participants that made it possible. Participants stated that their personal lived experiences allowed them to take action together, and made it possible to believe that each worker on-site could play their role in a collective effort to create change:

*That is why I’m here, that’s why I’m a union delegate. When I was doing it tough, I rang my boss and told him. He rang the union organiser and told him what happened to me. The organiser came straight to me and gave me comfort. He put me onto MATES, and they got me counselling, financial advisors, you name it. Nine months later I was all good and two years later I was a Union delegate, advanced first aid, safety representative at work and ended up with MATES as an ASIST worker* [A senior volunteer role in the MATES program]. (Steel Fixer, Male)

Participants had a strong sense of responsibility towards the industry and their peers, to do what was possible to reduce suicide and improve mental health in the industry. They described an industry where suicide had almost become normalized, where it was unspoken but accepted that occasionally workers were lost to suicide. Because of this, they felt that it was their responsibility to act and to make a difference to break down this expectation. This feeling became even stronger among participants who had contact with young workers who died by suicide, and in particular apprentices. The industry apprenticeship system creates obligations on tradespeople to not only teach apprentices the skills of the industry but also bring the young people into the industry culture. Participants highlighted how this would involve changing old ways and industry culture to foster a more open and direct discussion around mental health and suicide.

*After losing a young man I knew very well I questioned myself about what more I could have done. I recognised that maybe if he knew more about my story then he might have told me more about his.* (Senior Construction Manager, Male)

## DISCUSSION

This qualitative study investigated the MATES in Construction suicide prevention programme in Australia. Guided by Social Identity Theory and the SIMCA the study aimed to understand why construction workers chose to volunteer and advocate for industry-based suicide prevention programmes, and how their worker identity, solidarity and relationships impacted their volunteering and advocacy for the programme. The results provide a number of points for discussion in relation to the research questions.

**Fig. 3:**
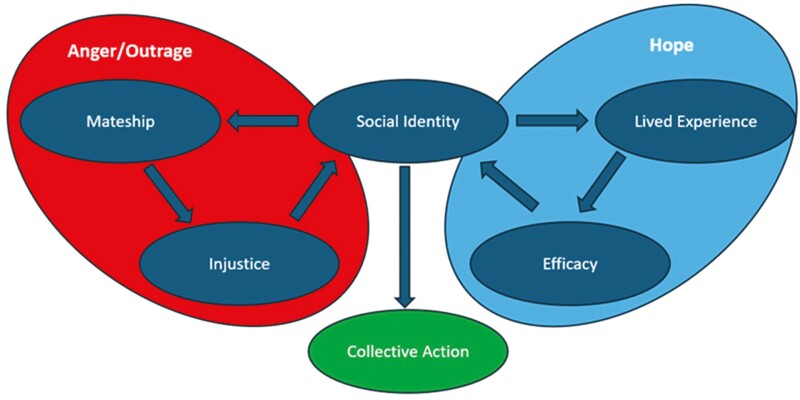
Illustration of the overlap between the SIMCA and the anger/outrage, hope and action models.

While the evidence about the impact of strong social identity on help-seeking in mental health and suicide prevention is mixed (Kearns *et al*., 2015; Ross *et al*., 2019; Baker *et al*., 2022), this study demonstrated possibility in using social identity to engage workers in suicide prevention for their community. The findings of this study aligned with the Social Identity Model of Collective Action (SIMCA) (Van Zomeren *et al*., 2008) and are consistent with the MATES anger/outrage, hope, action engagement model (LaMontagne and Shann, 2020). The overlap between these two models is represented in Figure 3. The SIMCA suggests that while injustice, efficacy and identity all predict collective action, social identity bridges injustice and efficacy as explanations of collective action. The strong social identity amongst construction workers facilitated mateship between workers. Mateship provided the lens through which high suicide rates were felt as an injustice leading to a sense or anger/outrage. Workers who also had lived and living experience not only saw the injustice but also saw their lived experience through their construction identity and therefore saw hope and efficacy in addressing the injustice collectively through MATES. The MATES programme was trusted because it is seen as a construction programme for the industry and by the industry, and this was an important factor in worker engagement in the programme. They highlighted the relatability of MATES staff as belonging to, and being part of, their industry as important.

While the construction industry is rapidly changing, there is still a sense of group identity, belonging and collective responsibility across the industry. However, it is important to note in the development of interventions with the construction industry, that there are also many sub-identities within the industry that can change as individuals move across different workplaces. The most common identities for participants in this study were as blue-collar workers, between union and non-union workers and between trades. Previous research has examined how these sub-identities may impact the ability of construction workers to recognize and understand important health and social issues (Heller *et al*., 2007; Milner *et al*., 2017; Ross *et al*., 2019; Greacen and Ross, 2023). The itinerant nature of work due to project-based employment means that workers continually renegotiate and moderate industry culture and norms as they move from job to job and thus create industry wide universal cultures. In the context of MATES, the industry-wide sense of mateship and obligations to such mateship unified participants across different identities. This obligation towards mates across the industry and the mobility of the workforce have provided unique diffusion opportunities for the MATES programme.

Lived experience of mental health crisis and/or suicidality was a significant motivator for engagement in workplace suicide prevention activities. This is consistent with previous research in the construction industry (Ross *et al*., 2019; King *et al*., 2023). Previous studies have suggested that the construction industry culture can be unsupportive of metal health conversations (Heller *et al*., 2007; King *et al*., 2023). However, this study was not consistent with previous findings. Participants generally found the industry had become much more supportive and that their sites generally were onboard with a suicide prevention focus. This could be due to MATES in Queensland having a much longer history and wider industry reach than other programmes. The impact of the programme on industry attitudes and behaviours may in turn support greater diffusion of the MATES programme. Such change is expected as a long-term outcome of the MATES programme in its programme logic model and could possibly be an outcome of 17 years of the MATES programme in the industry (LaMontagne and Shann, 2020).

Participants in the study identified mateship as an important part of the industry and part of their identity as construction workers. The feeling of obligation through mateship created a duty to act to protect the group once confronted with the knowledge of the impact of suicide on their industry. Mateship could be a mediating influence on the potential negative impact of masculine cultures as found in the construction industry on mental health. This finding is consistent with Sharp *et al.*, (2023), who identified what appears to be a misalignment in masculine values on one hand focusing on self-reliance and strength, and on the other teamwork and comradery. The mediator of these values is mateship valuing qualities such as friendship, loyalty and equality. Mateship is associated with selflessness, belonging and mutual help. While some have argued that new forms of mateship are less based on group membership and norms, and are more individualized and transactional (Butera, 2008), this study found that concepts of mateship were very much grounded in a collective ‘construction’ identity.

This study identified that there was a desire from volunteers to be part of reducing suicide rates in the construction industry. The simplicity of the MATES programme was significant in engaging workers in the programme. These findings are consistent with previous studies, which found that workers appreciated and valued being provided with knowledge, skills and abilities to be part of developing a safer industry (Ross *et al*., 2019; King *et al*., 2023) This study further identified lived experience and getting the opportunity to intervene as important in volunteer engagement. The high level of lived and living experience in this study may be an indication of lived experience being a facilitator for volunteering in the MATES programme, but also a unifying factor in the identity of volunteers and a sense of collective action and responsibility.

The findings of this study will have applicability to other situations where predominantly male cultural groups may be engaged in public health prevention campaigns through their sense of belonging and social identity. While this study examined why construction workers engaged with the MATES programme, further research is required to understand how this willingness to engage translates into actual collective action on worksites and programme diffusion across industry.

## CONCLUSION

This study found that the strong and specific construction industry identity of the MATES suicide prevention programme was an important factor in workers trusting and engaging with the programme. Workers had a strong social identity as construction workers and saw high suicide rates in the construction industry as an industry problem to be solved collectively. This study has shown that social identity and the bonds created between individuals within an identified social group, combined with motivation through lived experience can support the diffusion of public health prevention programmes. Further research into this in other settings is recommended, as is further research to better understand how workers are engaged with the programme and its longer-term impact on industry culture. This research has demonstrated the potential in utilizing social identity as a driver for diffusion of public health prevention programmes in predominantly male populations sharing a specific social identity.

## Supplementary Material

daae140_suppl_Supplementary_Material

## Data Availability

The data associated with this manuscript cannot be shared to protect the confidentiality of participants.

## References

[CIT0001] Australian Government. (2023) Jobs and Skills Australia; Labour Market Update, February 2023, Australian Government, Australia.

[CIT0002] Baker, C., Strong, R., McCord, C. and Redwine, T. (2022) Seeking support for mental health: evaluating social identity, social capital, and self-stigma of agricultural producers and their help-seeking preferences. Advancements in Agricultural Development, 3, 57–69.

[CIT0003] Bandara, P., Page, A., Reifels, L., Krysinska, K., Andriessen, K., Schlichthorst, M. et al. (2024) Attributable risk of suicide for populations in Australia. Frontiers in Psychiatry, 14, 1285542. 38260778 10.3389/fpsyt.2023.1285542 PMC10800872

[CIT0004] Braun, V. and Clarke, V. (2021) Can I use TA? Should I use TA? Should I not use TA? Comparing reflexive thematic analysis and other pattern‐based qualitative analytic approaches. Counselling and Psychotherapy Research, 21, 37–47.

[CIT0005] Braun, V. and Clarke, V. (2022) Thematic Analysis: A Practical Guide. Sage, London.

[CIT0006] Braun, V. and Clarke, V. (2024a) A critical review of the reporting of reflexive thematic analysis in Health Promotion International. Health Promotion International, 39, daae049. 38805676 10.1093/heapro/daae049 PMC11132294

[CIT0007] Braun, V. and Clarke, V. (2024b) Supporting best practice in reflexive thematic analysis reporting in palliative medicine: a review of published research and introduction to the Reflexive Thematic Analysis Reporting Guidelines (RTARG). Palliative Medicine, 38, 608–616, 10.1177/02692163241234800. 38469804 PMC11157981

[CIT0037] Butera, K. J. (2008) `Neo-mateship' in the 21st century. Journal of Sociology, 44, 265–281.

[CIT0008] Charmaz, K. (2017) The power of constructivist grounded theory for critical inquiry. Qualitative Inquiry, 23, 34–45.

[CIT0009] Dattani, S., Rodés-Guirao, L., Ritchie, H., Roser, M. and Ortiz-Ospina, E. (2023) Age-Standardized Suicide Rate - Sex: Both Sexes. World Health Organization, Global Health Observatory [original data]. *Our World Data*.

[CIT0010] Dearing, J. W. (2009) Applying diffusion of innovation theory to intervention development. Research on Social Work Practice, 19, 503–518. 20976022 10.1177/1049731509335569 PMC2957672

[CIT0011] Doran, C. M., Ling, R., Gullestrup, J., Swannell, S. and Milner, A. (2016) The impact of a suicide prevention strategy on reducing the economic cost of suicide in the new south wales construction industry. Crisis, 37, 121–129. 26695869 10.1027/0227-5910/a000362 PMC4901996

[CIT0012] Greacen, P. and Ross, V. (2023) Exploring the impact of social identity on the bullying of construction industry apprentices. International Journal of Environmental Research and Public Health, 20, 6980. 37947538 10.3390/ijerph20216980 PMC10649940

[CIT0013] Gullestrup, J., King, T., Thomas, S. L. and LaMontagne, A. D. (2023) Effectiveness of the Australian MATES in construction suicide prevention program: a systematic review. Health Promotion International, 38, daad082. 37647522 10.1093/heapro/daad082 PMC10468011

[CIT0014] Gullestrup, J., Lequertier, B. and Martin, G. (2011) MATES in construction: impact of a multimodal, community-based program for suicide prevention in the construction industry. International Journal of Environmental Research and Public Health, 8, 4180–4196. 22163201 10.3390/ijerph8114180 PMC3228565

[CIT0015] Heller, T. S., Hawgood, J. L. and De Leo, D. (2007) Correlates of suicide in building industry workers. Archives of Suicide Research, 11, 105–117. 17178646 10.1080/13811110600992977

[CIT0016] Hogg, M. A. (2016) Social identity theory. In McKeown, S.., Haji, R. and Ferguson, N. (eds), Understanding Peace and Conflict Through Social Identity Theory: Contemporary Global Perspectives. Springer, Switzerland.

[CIT0017] Kearns, M., Muldoon, O. T., Msetfi, R. M. and Surgenor, P. W. G. (2015) Understanding help-seeking amongst university students: the role of group identity, stigma, and exposure to suicide and help-seeking. Frontiers in Psychology, 6, 1462. 26483722 10.3389/fpsyg.2015.01462 PMC4586350

[CIT0018] King, K. E., Liddle, S. K. and Nicholas, A. (2023) A qualitative analysis of self-reported suicide gatekeeper competencies and behaviour within the Australian construction industry. Health Promotion Journal of Australia, 35, 10.1002/hpja.815. 37793646

[CIT0019] Krishnamoorthy, S., Mathieu, S., Armstrong, G., Ross, V., Francis, J., Reifels, L. et al. (2024) Implementation of complex suicide prevention interventions: insights into barriers, facilitators and lessons learned. Archives of Suicide Research, 20, 1–24. 10.1080/13811118.2024.2368127 38900080

[CIT0020] LaMontagne, A. D. and Shann, C. (2020) Mates in Construction Workplace Suicide Prevention Program: Articulation of Program Logic. MATES, Brisbane. https://mates.org.au/programlogic.

[CIT0021] Llamocca, E. N., Yeh, H. -H., Miller-Matero, L. R., Westphal, J., Frank, C. B., Simon, G. E. et al. (2023) Association between adverse social determinants of health and suicide death. Medical Care, 61, 744–749. 37708352 10.1097/MLR.0000000000001918 PMC10592168

[CIT0022] Maheen, H., Taouk, Y., Lamontagne, A. D., Spittal, M. and King, T. (2022) Suicide trends among Australian construction workers during years 2001–2019. Scientific Reports, 12, 20201. 36424429 10.1038/s41598-022-24575-x PMC9686251

[CIT0023] Martin, G., Swannel, S., Milner, A. and Gullestrup, J. (2016) MATES in construction suicide prevention program: a five year review. Journal of Community Medicine and Health Education, 6, 465.

[CIT0024] MATES in Construction. (2024) Suicide Prevention in the Construction Industry. MATES_in_Construction. https://www.mates.org.au/.

[CIT0025] Milner, A., Maheen, H., Currier, D. and LaMontagne, A. D. (2017) Male suicide among construction workers in Australia: a qualitative analysis of the major stressors precipitating death. BMC Public Health, 17, 1–9, 10.1186/s12889-017-4500-8 28629352 PMC5477155

[CIT0026] Milner, A., Morrell, S. and LaMontagne, A. D. (2014a) Economically inactive, unemployed and employed suicides in Australia by age and sex over a 10-year period: what was the impact of the 2007 economic recession? International Journal of Epidemiology, 43, 1500–1507. 25064642 10.1093/ije/dyu148

[CIT0027] Milner, A., Page, A. and LaMontagne, A. D. (2014b) Cause and effect in studies on unemployment, mental health and suicide: a meta-analytic and conceptual review. Psychological Medicine, 44, 909–917. 23834819 10.1017/S0033291713001621

[CIT0028] Milner, A., Spittal, M. J., Pirkis, J. and Lamontagne, A. D. (2013) Suicide by occupation: systematic review and meta-analysis. The British Journal of Psychiatry, 203, 409–416. 24297788 10.1192/bjp.bp.113.128405

[CIT0029] Neis, B. and Neil, K. (2020) Mental health in the construction industry: an interview with Australia’s MATES in construction CEO, Jorgen Gullestrup. Labour & Industry, 30, 413–429.

[CIT0030] Rogers, E. M. (2002) Diffusion of preventive innovations. Addictive Behaviors, 27, 989–993. 12369480 10.1016/s0306-4603(02)00300-3

[CIT0031] Ross, D. V., Caton, N., Gullestrup, J. and Kolves, K. (2019) Understanding the barriers and pathways to male help-seeking and help-offering: a mixed method study of the impact of the MATES in Construction program. International Journal of Environmental Research and Public Health, 16, 1–12, 10.3390/ijerph16162979 PMC6720173 31430939

[CIT0036] Sharp, P., Oliffe, J. L., Bottorff, J. L., Rice, S. M., Schulenkorf, N. and Caperchione, C. M. (2023) Connecting Australian masculinities and culture to mental health: Men’s perspectives and experiences. Men and Masculinities, 26, 112–133.

[CIT0032] United Nations. (2015) Transforming Our World: The 2030 Agenda for Sustainable Development. UN.

[CIT0033] Van Zomeren, M., Postmes, T. and Spears, R. (2008) Toward an integrative social identity model of collective action: a quantitative research synthesis of three socio-psychological perspectives. Psychological Bulletin, 134, 504–535. 18605818 10.1037/0033-2909.134.4.504

[CIT0034] WHO. (2021a) Live Life: An Implementation Guide for Suicide Prevention in Countries. World Health Organisation, Geneva.

[CIT0035] WHO. (2021b) Suicide Worldwide in 2019: Global Health Estimates. World Health Organisation, Geneva.

