# Supplementary material for: The role of social identity in a suicide prevention programme for construction workers in Australia
Source: Health Promot Int. 2024 Oct 22;39(5):daae140. doi: 10.1093/heapro/daae140 (PMC11495217; doi:10.1093/heapro/daae140)
Supplement: daae140_suppl_Supplementary_Material [file daae140_suppl_supplementary_material.docx]

| **Theme 1** | **A sense of belonging, connection and solidarity with industry and colleagues** |
| --- | --- |
| **Codes** | **Quote** |
| A sense of belonging | “We stand up for each other. We look after each other, we keep an eye out for each other.” (HSEQ Manager, Male) |
| The Bond between construction workers | *“As a tradie it is just the perfect scenario. To be able to make a difference in something that is important to me.” (Field Officer, Male)*  *The other day I was at the chemist and there was an old guy who would have been 65/70 with a CFMEU shirt on and I said hi mate how you going, I like your shirt. And I ended up sitting in the chemist talking to him for half an hour talking about our struggles that we’ve had over the years and what job we worked on and you know people that we knew. Like I say it’s a bit of a community and everyone knows someone right at the end of the day? (Delegate, Male)*  *To someone that’s not in the culture how do I explain to them what the brotherhood is?* (Steel fixer, Male) |
| The industry is relational | *“Relationships are everything in the industry. It is the relationship with the porta-loo guy I have used for 10-12 years now. I know if I call and say I need a porta-loo dropped off in the city at 2 am that it will happen.” (Health, Safety and Environment Manger, Male)* |
| Industry conditions creating connection | “*Some of these blokes, they’re away from home 12, 13 hours a day, you know? They leave on dark and get home on dark, and it can put a strain on their relationships*. *The other parents, have done the school run or the dinners or the house, it can cause friction, as crazy as it sounds.”* (Union Delegate, Female)  *“I think there definitely is a strong camaraderie in our industry. We spend so much time together, more time than we spend with our family at home. It makes it worthwhile getting to know the people onsite, to understand what they do and go through.”* (Dogman, Male) |
| Rough Industry | *That it’s industry-specific, that they are designed for our industry and they understand the high-risk industry that we work in on a daily basis and the pressures involved, obviously. (Dogman, Male)* |
| Intra-group status | *“But then I think compound that, there certainly is a pecking order in worksites and sometimes a lot of men feel that they can’t show too much weakness. The construction side is always about image and projection. It’s not an area for the faint-hearted. Then I think you combine that with the fact that you’ve got to be careful with what you say because if you say the wrong things to your superiors, it either sends a message that you can’t cope or it sends a message that somehow you’re weak or what have you.”* (Safety/Logistics Manager, Male) |
| Connecting through work history | *“If you notice someone with a hi-vis shirt on, you note the company and you will just talk to them. – “Oh, Billy, I worked with him on the Queen Street job”. You find common ground. It’s very easy.”* (Dogman, Male)  *“At the chemist is as and old guy with a CFMEU (Union) shirt. I ended up talking to him for half an hour about our struggles over the years, what jobs we had worked on and who we knew. It is a bit of a community, and everyone knows someone you know.”*  (Union Delegate, Male) |
| Solidarity across industry | *If you’re a sub-contractor and you’re working for yourself and you’ve got a couple of boys working for you, you’re not going to – I mean you might be mates with these guys but you’re also there to make money. At the end of the day that’s all we’re there for, but I think you’ve got the best intentions a lot of the time you know.* (Union Delegate, Male)  *Yeah 100%, that’s one thing about MATES is it’s not just for the workers right, I’m sure that supervisors and foremen and stuff utilise it as well. They go through the similar struggles that we do; probably more so because they don’t have that network and that family and that support and they’re doing the big hours and they’re probably a bit isolated.* (Delegate, Male) |
| Impact of union and industrial struggle | *Within our industry too we’ve had our struggles and if we weren’t having these struggles together, we probably wouldn’t have a lot of the things that we have now you know. So, the mateship is what brings us together and keeps us together and moulds us to have the fights that we need to have.* (Union Delegate, Health and Safety Representative Male) |
| Itinerant nature of work | *I think the other thing it does have is it probably has a boom, bust type. I think we still have that and so we have people who build up expectations and lifestyles and that sort of thing when times are good and then when we get to times a bit like now they struggle because they can’t afford the jet skis and the trailbikes plus the boat and the new car and all the rest of it which they were used to and unless you actually realise that that doesn’t make me a failure, it’s just that we’re going through those cycles* (Engineering Manager, Male) |
| Workers helping to diffuse the program | *“We went to [Provincial town] and we were struggling to get MATES access to the site because [the global project management company], being who they are. At that stage we were working for [Provincial town] Ports on the seawall. We needed to be doing MATES on the site and we basically went against [the global project management company] and brought you in behind their back. I think for me I think that was something that I had an opportunity to do and we were able to manipulate the situation for good. I think for me they’re the things which I feel proudest of. (Engineering Manager, Male)*  *I think MATES is one of the things you can take from site to site and you can share your experiences with and you can talk to people and say, “Are you guys aware that there is a MATES in Construction people out there? There is people you can talk to.” I think the word gets spread around that way and that’s where I think MATES reflects out into a lot of areas. In a lot of ways, it would be good if it could find itself into other areas too. I don’t know what MATES are working on, but even the hospitals and stuff like that. But yeah, that’s how I tend to think. It’s become universally – it’s nearly like what Holden cars were like some years ago where they touched everybody to some extent.* (Safety / Logistics Manager, Male) |
| **Theme 2** | **While identities change between work roles and context while there was an overall sense of identity and solidarity across the sector with MATES.** |
| Sub-identities | *To me, that is what makes MATES stand out, and no one is ever any better than the other. So, we have hierarchies in the industry, right? From management, CEOs, whatever. But no one is better than anyone else. Despite those role descriptions and delineation between levels, et cetera, every human is considered to be equal. I think that’s why it works.* (Senior Lecturer, Female) |
| Contrast between in and out groups | *You’ve got obviously the blue collar and the white collar, it’s a bit different and I think sometimes the white collar is even less likely to talk because they’re seen to be the ones who should have the answers*. (Engineering Manager, Male)  *There is mateship in the construction industry but it’s mainly in the union industry. When you go out into the non-financial world away from the union, it’s pretty much every man for themselves. They sure have some form of mateship. But the union bring everyone together. They show people mateship. A lot of people from the non-EBA sector that come into the EBA sector, that is the first thing that they see, the brotherhood, the love, they get looked after. Out there they don’t get looked after – you know it’s like wild animals in the bush. You know what I mean?* (Steel Fixer, Male*)* |
| Having each other’s back | *“I don’t appreciate foremen talking to workers in a certain way. I warn the foremen that if they don’t improve the workers will finish the job while they are down the road. I love doing that.” (Dogman, Male)* |
| MATES as part of the in-group | *“Before there wasn’t really anything around, people talked, but they talked to outsiders of the industry. It is not just kept in-house. It says Mates in Construction, it’s construction only.”* (Union Delegate, Female)  *I suppose I don't want to just keep going on, but can I say the way that the program works it dovetail into the industry. The way the MATES program work is there is no ego’s and no bullshit – it just fits in. (Health, Safety, Environment and Quality Manager, Male)*  *And that’s what makes it different, you know MATES is not – you don’t feel like you’re going to an institution. You’re just having a chat you know. You might not even know that it’s helping you, but it is.* (Delegate, Male)  *“It is not just some random person that is a jack of all trade’s kind of thing. MATES are specifically for construction, and I think guys relate to that.* (Carpenter, Male) |
| Impact of globalisation – the non-Australian owners | *The developers have all the power and the entire construction process contract process and everything else, like that.[….] I think the relationship from developer, to builder, to subcontractor is where the developer holding all the strings and seems to be the all-powerful one. They probably need to be better policed from an accountability sort of thing. I think starting at the top will work all the way through and will give better mental health out comes across the industry. (Senior Construction Manager)* |
| The loss of career path from bottom to top | *We look out for each other. The fact that we now have corporate management that doesn’t come from that cultural background, I think they struggle to understand a little bit and I think they definitely have a bit more of a mindset that you do what you’re told as opposed to you think for yourself and you actually make a difference by what your behaviours and your cultures are.* (Engineering Manager, Male) |
| Creating a difference in our industry | *“I needed to be involved because of the rate of suicide, particularly in the construction industry and in particular male suicide.”* (Health and Safety Coordinator, Male) |
| **Theme 3** | **The role of industry mateship in engaging in suicide prevention** |
| Mateship as a cultural value | *I don't think we're losing the mateship per se; I think it is a lot stronger in construction. You spend all day with the boys around you so, - you've got a sort of different relationship with the boys around you than you do the, you know, boys you grew up with or mates at home or mates down the pub*. (Foundation Labourer, Male) |
| Glass ceiling for mateship | *“I think there definitely is connection amongst the fluoro guys, the real blue-collar workers and I think that stops at a management point.* (Site Manager, Male) |
| Australian culture and mateship | *“It is what Aussies are about. It is all about being mates and that.”* (Health and Safety Representative, Male)  *“Mates is such an Australian word that goes back forever to the convict days. We had to rely on each other to survive and to break out together. Mates look after mates, that is really key.”* (Health, Safety and Environment Manger, Male) |
| Creating rules for engagement | *“To be a man is to be able to reach out to men and to reach out for help and to receive help that is one of the key foundations.”*  (Health and Safety Coordinator, Male) |
| General Awareness Training speaking to mateship and industry obligation | *“I suppose just the initial GAT training, because the person we have on board delivers that very well and they’re very passionate about it and it’s very real and it’s coming from their heart and probably in some instances through personal experiences and it’s a strong message and once you’ve finished the GAT training, you think, “I’ve got to keep going with this.” (Head of Health, Safety, Environment, Quality and Training, Male)*  *So we got that started and then it really hit a point with me with General Awareness Training – it was really weird. It was like when it was happening, it was like there was no-one else in the room, they were just talking to me, sort of thing. Do you know what I mean? (Head of HSEQT, Male)* |
| Duty to offer and accept help | *“I think it just reinforce that we’ve just got to do something, and you can’t just stand back and wait or watch. In life there are times when you got to jump the fence and participate and for me this is just one of those things where you got to do what you can.”* (Engineering Manager, Male) |
| Belonging to a social class | *“I grew up in the housing commission and a lot of us come from a lower socio economical background. We didn’t do well at school. This is why it resonates.”* (Health, Safety and Environment Manger, Male) |
| Mateship and adversity | *“I just felt a lot of our fellow colleagues out there needed a bit more and that is when I started to get involved with MATES.”* (Safety/Logistics Manager, Male) |
| Mateship obligations through struggle | *“Within our industry we have had our struggles. Without these struggles we probably would not have a lot of the conditions we have. Mateship is what brings us together and mould us to have the fights we need to have.”* (Union Delegate, Male) |
| Blue-collar mateship superiority | *I think that there’s definitely a connection amongst like the fluoro guys – the real blue-collar workers and I think that in some ways that stops at a management point. It becomes – my role is far more judicial and administrative which can create more conflict that amongst the direct workers.* (Site Manager, Male) |
| Mateship alignment with trade union language | *“We have the Unions and they use very strong language like “comrade” and “brother”. They are strong because they build strong connections between workers. I can walk down the street and recognise a construction worker, perhaps because of the name on his shirt, and I will say hi because I feel I know him because we are in the same industry*.” (Health, Safety and Environment Manger, Male)  *“I think a lot of it is to do with the union as well, the fact that the majority of people are in the union and the union promote family orientation and solidarity and MATES promotes mateship.”* (Health and Safety Representative, Male) |
| Mates similarity with trade union structures | *“Why do we wear a union shirt? Why do we wear our football team shirt? The MATES brand makes it recognised throughout the industry. […] You know, it is so important, MATES is so important. It is a symbolisation of our industry.”* (Plumber/Operator, Male) |
| Compatibility with union delegate role | *My brother is the CFMEU delegate at [project name] right now. So he’s with MATES in construction most days I think by the sounds of some of the things he's dealing with. (Carpenter, Male)*  *I’ve never been aware, I guess, of the state of mental health until I got involved in health and safety obviously and the delegate’s role. I was never aware of how bad it was. (Dogman, Male)* |
| Becoming a better delegate through MATES skills | *I’d advise anybody to become a union delegate to make sure that they do their Connector’s course.* (Health and Safety Representative / Union delegate, Male)  *I think it should be part of the delegate’s role, personally. I mean, once you do step into that area, you are pretty much, for want of a better word, you’re probably a babysitter. You’re probably a social worker, so to speak, sometimes.* (Dogman, Male*)* |
| **Theme 4** | **Lived Experiences Combined with mateship and collective responsibility provided hope for change** |
| Living with mental health struggles in the industry | *“I got diagnosed with disassociation. I had a bit of a rough upbringing. I’ve got the little-big syndrome you know. Little Mick is trying to hold Big Mick back. I just make jokes about it now because it’s the best way for me to cope with it.”* (Health, Safety and Environment Manger, Male)  *“It’s a journey for me. I have good and I have tough days. The beauty of bipolar is that some of those good days are a little too good, a little manic. The bad days can be challenging and that can be pretty tough as well.”* (Field Officer, Male) |
| Impact of different experiences | *“Tragically we have had a sub-contractor there on Friday and then not on Monday – in my time and circle it has happened three times over 10 years. If we don’t do what we can and ask how are you really? Then potentially we leave site on Friday and the person may not be there on Monday.” (Health, Safety and Environment Manager, Male)*  *“I was helping one person and I talked to him the night before and he didn’t sound right. I rang his father and said: We need to catch up with him for a coffee tomorrow. He’s not in a good place. Unfortunately, we never got to have that coffee with him because he took his life.”* (Health, Safety and Environment Manager, Male)  “*One particular one comes to mind. He was a young apprentice about 16 years old and his father took his life by suicide. That really touched me, you know, his father was his role model. He absolutely idolised him. I thought how that’s going to affect that young fellow for the rest of his life”* (Field Officer, Male) |
| It is difficult to ask for help | *“I even remember Googling some mental health places because a couple of nights it was affecting me a lot. I remember looking at my phone, but I never called them. I went home, had a week off, came back and grabbed my stuff and just left.” (Carpenter, Male)* |
| Feeling impowered to make a difference | *I guess that is why the program resonates with me personally, but also why I think it resonates with the industry. When we talk about strengths of the program and why it resonates and the language and all that, but at it’s essence it is about empowerment. If we can empower people, then they will do the right things whether it is to use the grinder correctly and stay physically safe or whether it is having a conversation with someone struggling and connecting them to help. If we empower people, they will do it.* (Health, Safety, Environment and Quality Manger, Male*)* |
| Be an example for others | *“During a very challenging season of my life after my marriage breakdown I found I had a choice. I can do it myself and be the big hero that can solve the whole problem myself or I can learn to reach out and allow others to reach out to me.”* (Health and Safety Coordinator, Male)  *I’m a functioning depressed person myself, so obviously I’ve got some serious values around maintaining a level of awareness and assistance throughout the company.* (Head of Health, Safety, Environment, Quality and Training, Male)  *As I say, what drives me is the fact that there was nobody for me to help or offer help when I probably needed it the most. I'm aware of what that can be like. I want to be a part of changing the culture of the industry because I've been through. You say lived experience; I've been there.* (Field Officer, Male) |
| Lived Experience makes you relatable | *I love it when [Name] and other field officers comes, they talk at our level. They know who we are because they know the industry.* (Health, Safety, Environment and Quality Manger, Male*)* |
| High suicide rates is an injustice to the industry | *So, you know, our industry… You know, I personally had a godson commit suicide a few years ago, was working on a construction site. So, it has touched my family twice. One was my uncle, and one was the godson. To think that there are other people out there that we could help, (HSEQ Manager, Male)*  *And we as a construction community we can recognise those things and get those people help, it’s going to be better for our industry. You know yourself, I don’t know what the statistics are exactly, but since you would have started MATES the suicide rates within construction would have dropped significantly I would have thought. (Delegate, Male)* |
| Responsibility to mates | *…maybe having car trouble in a car park and he might have walked from fucking four or five jobs down and he's come over to his car and his car's flat. Yeah 100 per cent. You see him in high VIZ regardless whether he works in commercial or residential, whatever, you - yeah, I would find myself always saying, "Hey, mate, are you all good there or what? Do you need a jump?" (Foundation Labourer, Male)*  *I never used to feel like this, and I used to never think like this. Until I had my bad experience, and the union come in and done what they done. And then I just seen it all. You know what I mean. I was like “Holy shit, I fucken – I want to be that for someone else.” And that’s how it come about. (Site Delegate)* |
| Responsibility to young workers | *“I have lost three young men I knew well through work in construction and rugby. They happened to take their own life and unfortunately one took his own life on a construction site in Sydney. It is pretty damn close to me.”* (Senior Construction Manager, Male) |
